# Seromuscular Colonic Flap for Intrapelvic Soft-Tissue Coverage: A Reconstructive Option for Plastic Surgeons When Traditionally Used Flaps Are Not Available

**DOI:** 10.1155/2015/563641

**Published:** 2015-11-24

**Authors:** Johnathon Aho, Sebastian Winocour, Ziyad S. Hammoudeh, Heidi Nelson, Peter Rose, Nho V. Tran

**Affiliations:** ^1^Division of Plastic Surgery, Department of Surgery, Mayo Clinic, 200 First Street SW, Rochester, MN 55905, USA; ^2^Division of Colorectal Surgery, Department of Surgery, Mayo Clinic, 200 First Street SW, Rochester, MN 55905, USA; ^3^Department of Orthopedic Surgery, Mayo Clinic, 200 First Street SW, Rochester, MN 55905, USA

## Abstract

*Background*. Reconstruction of intrapelvic defects can be a challenging problem in patients with limited regional muscle flap options and previously resected omentum. In such situations, alternative methods of mobilizing vascularized tissue may be required.* Methods*. A case of a patient that underwent pelvic extirpation for recurrent rectal cancer who had limited donor sites for flap reconstruction is presented. The mucosa was removed from a blind loop of colon, and a pedicled seromuscular flap based on the colonic mesentery was placed into the pelvis for vascularized soft-tissue coverage and elimination of dead space.* Results*. The postoperative course was only complicated by a small subcutaneous fluid collection beneath the sacrectomy skin incision, which was drained with radiological assistance. The patient recovered without any major postoperative complications.* Conclusion*. Seromuscular colonic flap is a useful option for soft-tissue coverage after pelvic extirpation and should be considered by plastic surgeons when other reconstruction options are not available.

## 1. Introduction

Pelvic extirpation for recurrent or locally advanced intrapelvic cancers often results in a large dead space defect. Patients remain at high risk for complications, such as perineal wound dehiscence, hernia, bowel obstruction, and abscess, which are further increased by neoadjuvant and/or adjuvant radiotherapy. Plastic surgeons have used a variety of flaps to fill the pelvic soft-tissue void following resection and provide vascularized tissue to aid in healing; common options for immediate flap reconstruction include the omentum, rectus abdominis, and gracilis muscles. However, in patients with recurrent disease, some or all of these flaps may not be available due to resection with the tumor, prior surgical scars, or use of the flap for previous reconstruction. A case of a patient with recurrent rectal cancer and limited flap options in which a seromuscular colonic flap was used to fill the large soft-tissue void following tumor resection is presented.

## 2. Report of a Case

**Figure 1:**
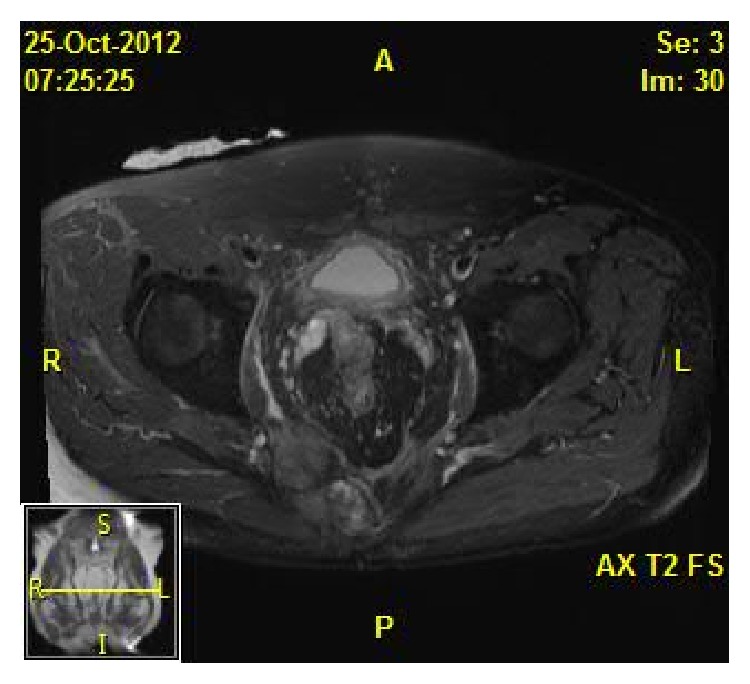
Biopsy-proven recurrent rectal adenocarcinoma invading the sacrum.

**Figure 2:**
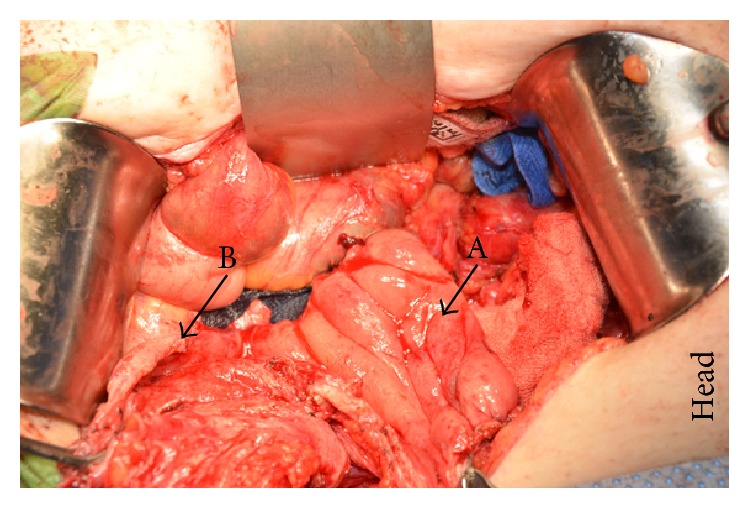
A blind loop of sigmoid colon preserved on native mesenteric blood supply after extirpation of pelvic mass. Sigmoid colon (A) and direction of pelvic defect (B).

The patient is a 57-year-old male with recurrent rectal carcinoma who underwent proctectomy with coloanal J-pouch anastomosis eleven years prior to presentation. The patient presented to an outside hospital with colonic obstruction. An abdominal and pelvic CT scan demonstrated an 8.5 × 7 cm soft-tissue mass in the presacral space with invasion of the sacrum and coccyx, at which point he was then referred to the authors' institution for a higher level of care. Image-guided biopsy confirmed recurrent rectal adenocarcinoma, and a metastatic workup was negative. He was initially treated with a temporizing diverting loop ileostomy to relieve the obstruction, which was followed by an outpatient course of chemotherapy and external beam radiation. Following completion of neoadjuvant therapy, he underwent magnetic resonance imaging, which confirmed a decrease in size of the rectal tumor to 6 × 3.5 × 4.3 cm with persistent sacral and coccygeal invasion (Figure 1). Staged surgical resection with intraoperative radiation to the pelvis was planned, involving a multidisciplinary team of colorectal, orthopedic, and plastic surgeons. En bloc tumor resection with distal sacrectomy was performed, leaving a large pelvic defect without involvement of the perineal skin. The colon was divided with a stapler just proximal to the previous anastomosis in order to be able to perform the dissection, leaving a blind loop of sigmoid colon that was preserved on its native mesenteric blood supply (Figure 2).

**Figure 3:**
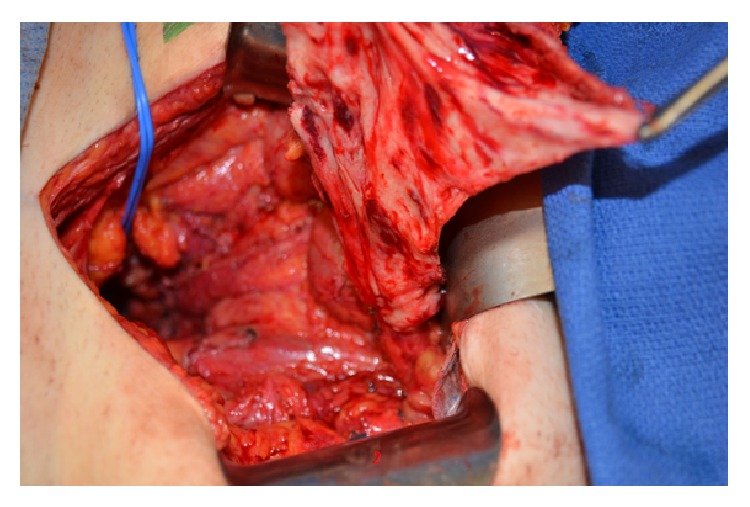
Seromuscular colon flap after mucosectomy with mesentery pedicle.

Multiple intrapelvic soft-tissue reconstructive options were considered but were not available or would not reach the defect. Both inferior epigastric vessels had been previously surgically divided by a right-sided ostomy site and a left-sided transverse abdominal scar, preventing use of the rectus abdominis as a pedicled muscle flap. No residual omentum was present due to multiple previous abdominal explorations and dense adhesions preventing dissection into the upper abdomen. The preserved blind loop of colon was opened along its antimesenteric border. A solution of 1% lidocaine with 1 : 200,000 epinephrine was injected into the submucosal plane to allow a nearly bloodless mucosectomy (Figure 3). The mesenteric pedicle supplying the fileted segment of colon was carefully dissected to increase the flap's length. The flap was inset with the muscular layer of colon facing the raw pelvic musculature. The flap was secured in place using absorbable sutures and provided sufficient volume to fill the pelvic dead space. The postoperative course was only complicated by a small subcutaneous fluid collection beneath his sacrectomy skin incision, which was drained with radiological assistance. The patient recovered without further postoperative complications.

## 3. Discussion

This case represents the unique use of a seromuscular colonic flap for soft-tissue reconstruction of a pelvic defect by plastic surgeons. A similar use of seromuscularis was described by a colorectal surgery group in Thailand in eight patients [1]. The seromuscular colonic flap can be tremendously useful in reconstructing intrapelvic defects when options are limited and should be considered as a viable option in the armamentarium of plastic surgeons performing complex pelvic reconstruction. The seromuscular colonic flap allows for use of vascularized tissue that would otherwise be discarded due to resection without the added donor site morbidity associated with a muscle flap. To utilize this flap, the defunctionalized colonic mucosa must be removed to avoid intraperitoneal mucus accumulation. As the colonic lumen is entered for generation of this flap, there is an increased possibility of stool contamination in the field and possible increased risk of subsequent abscess and sepsis. Care should be taken to limit gross contamination of the pelvic cavity with stool and a bowel preparation should be considered preoperatively. Preoperative communication and coordination with the oncologic surgeons is essential; the oncologic surgeons must be made aware preoperatively of the possible use of this flap so that they do not inadvertently divide the mesenteric blood supply or resect the blind colonic loop entirely before the plastic surgeon enters the operating room.

The benefits of soft-tissue reconstruction after pelvic extirpation for locally advanced rectal cancer have been well reported [2–4]. The vertical rectus abdominis musculocutaneous (VRAM) flap is often preferred for immediate perineal and neovaginal reconstruction over the gracilis muscle flap [5, 6]. However, use of abdominal tissue does have limitations, including difficulty creating an end-stoma and increased incidence of postoperative hernia [3, 5].

While muscle flap techniques are widely used and have been reported for pelvic extirpation, a colonic seromuscular flap is also locally available and should be considered. Use of a defunctionalized bowel segment, which under normal circumstance is resected and discarded, takes advantage of the spare part concept to minimize morbidity. Bowel mucosal flaps have been well established for closure of vaginal defects, gender reassignment surgery, and treatment of vaginal agenesis in which secretory tissue is desired for lubrication [7, 8]. However, use of the colon's seromuscular layer can be extremely beneficial to patients in which nonsecretory vascularized tissue is needed when there are limited reconstructive options following pelvic extirpation. A limitation of this flap's utility for reconstruction is the size of the pelvic defect and lack of squamous epithelia which are considerations for larger pelvic reconstructions. In cases where redundant colonic tissue is available and other options are not available, the seromuscular colonic flap should be considered for soft-tissue reconstruction of pelvic defects.
